# A formulation for asphalt concrete air void during service life by adopting a hybrid evolutionary polynomial regression and multi-gene genetic programming

**DOI:** 10.1038/s41598-024-61313-x

**Published:** 2024-06-10

**Authors:** Ali Reza Ghanizadeh, Amir Tavana Amlashi, Alireza Bahrami, Haytham F. Isleem, Samer Dessouky

**Affiliations:** 1 Department of Civil Engineering, Sirjan University of Technology, Sirjan, Iran; 2 School of Civil and Environmental Engineering and Construction Management, University of Texas at San Antonio, 1 UTSA Circle San Antonio, San Antonio, TX 78249 USA; 3 Department of Building Engineering, Energy Systems and Sustainability Science, Faculty of Engineering and Sustainable Development, University of Gävle, 801 76 Gävle, Sweden; 4 School of Applied Technologies, Qujing Normal University, Qujing, 655011 Yunnan China

**Keywords:** Air void, Service life, Asphalt concrete mixture, Evolutionary polynomial regression, Teaching–learning based optimization algorithm, Genetic programming, Engineering, Civil engineering

## Abstract

Bitumen, aggregate, and air void (VA) are the three primary ingredients of asphalt concrete. VA changes over time as a function of four factors: traffic loads and repetitions, environmental regimes, compaction, and asphalt mix composition. Due to the high as-constructed VA content of the material, it is expected that VA will reduce over time, causing rutting during initial traffic periods. Eventually, the material will undergo shear flow when it reaches its densest state with optimum aggregate interlock or refusal VA content. Therefore, to ensure the quality of construction, VA in asphalt mixture need to be modeled throughout the service life. This study aims to implement a hybrid evolutionary polynomial regression (EPR) combined with a teaching–learning based optimization (TLBO) algorithm and multi-gene genetic programming (MGGP) to predict the VA percentage of asphalt mixture during the service life. For this purpose, 324 data records of VA were collected from the literature. The variables selected as inputs were original as-constructed VA, ${VA}_{orig}$ (%); mean annual air temperature, $MAAT$ (°F); original viscosity at 77 °F, ${\eta }_{orig,77}$ (Mega-Poises); and $time$ (months). EPR-TLBO was found to be superior to MGGP and existing empirical models due to the interquartile ranges of absolute error boxes equal to 0.67%. EPR-TLBO had an R^2^ value of more than 0.90 in both the training and testing phases, and only less than 20% of the records were predicted utilizing this model with more than 20% deviation from the observed values. As determined by the sensitivity analysis, ${\eta }_{orig,77}$ is the most significant of the four input variables, while *time* is the least one. A parametric study showed that regardless of $MAAT$, ${\eta }_{orig,77},$ of 0.3 Mega-Poises, and ${VA}_{orig}$ above 6% can be ideal for improving the pavement service life. It was also witnessed that with an increase of $MAAT$ from 37 to 75 °F, the serviceability of asphalt concrete takes 15 months less on average.

## Introduction

Bitumen, aggregate, and air void (VA) are the three primary ingredients of asphalt concrete^1^. Insufficient VA leads to macro cracks from repetitive load stress^2^, highlighting its importance in pavement stability. Each percent reduction in VA decreases the pavement’s ability to resist the deformation under traffic and various weather conditions by five points, underscoring its pivotal role in the strength and durability of asphalt materials^3,4^. Low VA values are linked to bleeding and rutting under repetitive traffic, causing a loss in serviceability^5,6^, whereas high VA values can result in excessive aging and stripping^7^, and reduce the fatigue life in moist conditions^8^. Studies consistently aim to predict the pavement life by examining these parameters, with findings suggesting that a 1% increase in VA can reduce the pavement’s lifespan by a year, whereas decreasing VA by 1% could extend it by 10%^9–11^. Further research, including LCCAs by Tran et al. demonstrated significant cost savings and service life extensions by managing VA levels, confirming the substantial economic and performance impacts of VA management under diverse climatic conditions^12–14^. VA in asphalt concrete noticeably influences its performance, affecting the thermal conductivity, specific heat capacity, and emissivity. High VA mixtures exhibit higher surface temperatures, underscoring the VA’s importance in asphalt quality and durability^15^. Furthermore, reduced VA can lead to pavement issues like bleeding, instability, and decreased skid resistance, which are critical for safety^16^. To address these challenges, researchers have developed predictive models for the VA content, enhancing the construction productivity and ensuring the longevity and functionality of pavement structures. He^17^ used traditional statistical methods to estimate the initial amount of VA in asphalt pavement, while Kassam et al.^18^ linked initial VA to the number of roller passes or the compaction index (CI). Kassem et al.^19^ further refined this approach by employing a compaction tracking system to establish the relationship between VA and CI. Advances in technology have also enabled the monitoring of asphalt mixture density through radar technology^20,21^, with improvements in VA percentage measurements being made utilizing a ground-penetrating radar (GPR) array system as demonstrated by Hoegh et al.^22^. Additionally, the distribution of VA within open-grade asphalt mixtures has been explored through x-ray computed tomography and image analysis^23–25^. Understanding the dynamics of VA after construction is crucial for predicting the pavement performance and potential failures. VA changes over time influenced by factors such as traffic loads, environmental conditions, compaction techniques, and asphalt mix composition^26^. These factors combine to determine the stability and durability of the pavement, emphasizing the importance of managing initial and subsequent VA levels accurately. Three regression models have been presented so far for predicting aged VA based on four input variables including ${VA}_{orig}$ original as-constructed VA (%); $MAAT$, mean annual air temperature, (°F); ${\eta }_{orig,77},$ original viscosity at 77 ºF (Mega-Poises); and $time$ (months).

The first regression model by Mirza et al.^27^ is given below:

1 $${\text{VA}}=\frac{V{A}_{orig}+0.0111\times time-2}{1+0.000424\times time\times MAAT+0.001169\times \frac{time}{{\eta }_{orig,77}}}+2$$

The second model proposed incorporating data from Mirza and NCAT^26^ is presented as follows:

2 $${\text{VA}}=\frac{V{A}_{orig}+0.0398\times time-2}{1+0.00065\times time\times MAAT+0.0000101\times \frac{time}{{\eta }_{orig,77}}}+2$$

It can be seen that Eqs. (1 and 2) have the same form; the only difference is in the constant coefficients, which emerged due to different data points utilized in calibrating the regression models.

Equation (3) presents the third suggested model by the NCHRP’s guide for mechanistic-empirical design of new and rehabilitated pavement structures^26^:

3 $${\text{VA}}=\frac{V{A}_{orig}+{exp}^{(-1.0528\times time)}-1}{1+0.01406\times time+0.00125\times {time}^{0.2307}\times MAAT-0.00325\times time\times {\eta }_{orig,77}}$$

Intelligent algorithms, particularly artificial neural networks (ANNs), are increasingly utilized in civil engineering to enhance predictive modeling^28^. Zhao et al.^16^ applied multiple machine learning methods, notably finding that support vector regression (SVR) most accurately estimated the initial VA, based on parameters like aggregate gradation and asphalt-aggregate ratio. Similarly, Zavrtanik et al.^1^ and Dalhat and Osman^29^ leveraged ANNs to analyze asphalt properties and predict specific gravity in asphalt mixes, respectively. Othman^30^ also used ANNs to determine key Marshall mix design parameters, highlighting the diverse applications of ANNs in optimizing asphalt mixture designs and improving material property predictions. Due to the black-box nature of ANN and its complex calculations which usually cannot be performed manually, engineers are always in need of new solutions that provide straightforward equations to predict unidentified characteristics and make it easy to predict the outcome of projects by placing new measured values^31,32^. As a result, symbolic regression methods like evolutionary polynomial regression (EPR) and multi-gene genetic programming (MGGP) have become very prominent and widely employed in recent years.

An extended regression method called EPR incorporates the advantages of both least square regression and genetic programming (GP) regression approaches^33^. EPR has effectively modeled various engineering challenges, such as the behavior of unsaturated soils, slope stability, soil permeability, asphalt flow number, and mechanical properties of stabilized sandy soil^34–41^. In this study, the teaching–learning based optimization (TLBO) algorithm, a population-based evolutionary method that emulates the teacher-student interaction, was used to refine the constants in EPR models, enhancing their accuracy and efficiency without the need for tuning parameters other than iteration count and population size^42,43^. Additionally, MGGP has been utilized as a machine learning technique to generate nonlinear predictive formulas efficiently. MGGP, founded on principles of genetics and natural selection by Koza^45–47^, leverages parse trees instead of fixed-length binary strings, providing the flexibility needed for complex problem-solving across various domains without being bound by prior data patterns^44,48^. This approach has addressed numerous engineering challenges effectively^49–55^.

By considering what has been briefly explained above, there is a gap in the development of models that can provide a simple and practical equation for predicting aged VA values more accurately, which can facilitate the design process of asphalt concrete, enhance its performance prediction, and finally, improve the safety of the roads. It is the goal of this study to address these gaps by: (I) compiling a comprehensive database of asphalt concrete VA during the service life utilizing published sources, (II) implementing hybridized EPR by applying the TLBO algorithm and MGGP for modeling VA, (III) comparing the models presented in this study to previously developed models, and (IV) identifying the importance of input variables on VA of asphalt mixture during its service life and conducting parametric study based on each inputs variation in a specific range and also predicting VA. Through this research, a further understanding of VA during the service life will be gained and it will pave the way for a more accurate simulation of asphalt concrete performance during the service life.

## Methodological background

### EPR

**Figure 1:**
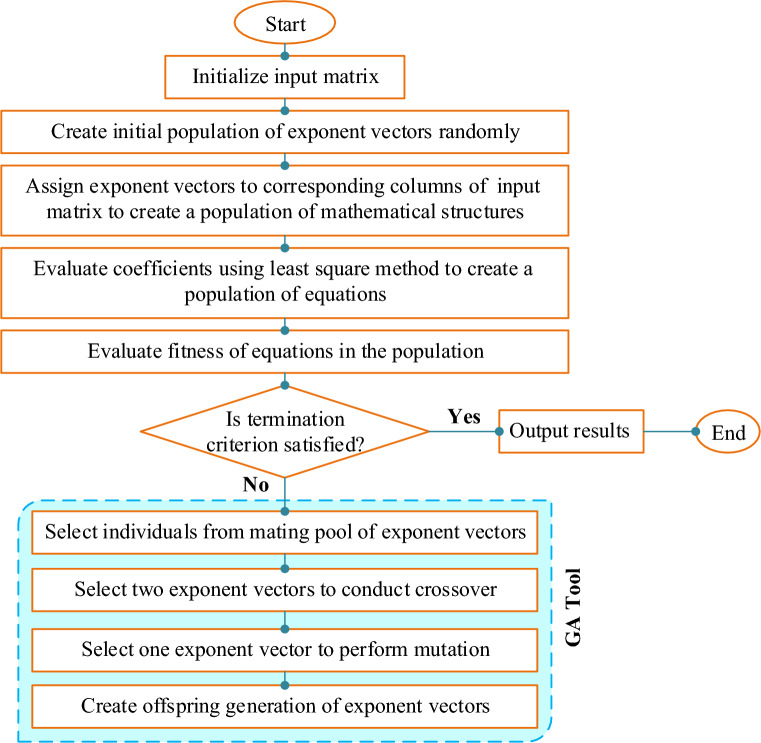
Flowchart for EPR process^64^.

Over the past two decades, advances in computational tools have considerably facilitated the use of computational intelligence techniques in fields like data mining and soft computing^33^. Among these techniques, EPR stands out due to its integration of traditional regression and GP elements^56^, making it effective for modeling complex environmental phenomena^38,57–60^. First introduced by Giustolisi and Savic^61^, EPR operates through a two-step process that constructs symbolic models by determining structure and refining relationships between inputs and outputs^62^. This method evaluates the accuracy of its output functions through the correlation coefficient and adjusts its models based on the desired accuracy and other criteria like the number of generations or sentences in the model^61^. The EPR method's efficacy lies in its ability to produce high-fit models quickly, employing polynomial relations with a limited number of sentences to avoid unnecessary complexity while maintaining accuracy^33^. Various forms of the equation are used to fit the data, involving different powers and user-defined transformations of the input variables to best model the relationships within the data. Each version of the equation demonstrates the flexibility of EPR in accommodating different data structures and analysis needs, with parameters adjusted to refine predictions and improve model fit^63^. This adaptive, iterative method allows EPR to remain robust across various applications, dynamically adjusting to new data and findings. Figure 1 displays the flowchart of the EPR method.

### TLBO

The TLBO algorithm, developed by Rao et al.^65^, is inspired by the educational influence of teachers on students in a classroom setting. TLBO operates in two main phases as the teacher phase and the student phase. In the teacher phase, the algorithm simulates a classroom where one "teacher" (the best solution) aims to improve the overall learning (performance) of a group of "students" (solutions). Each student’s performance on various "subjects" (design variables) is evaluated, and the best performing student’s results influence the rest of the population. The key mathematical representation of this phase involves adjusting each student’s solution based on the difference between the best solution and the class average, modulated by a random factor (r_i_) and a teaching factor (T_F_), which determines the magnitude of learning:

4 $${Difference\_Mean}_{j,k,i}={r}_{i}\left({X}_{j,kbest,i}-{T}_{F}{M}_{j,i}\right)$$

5 $${X}_{j,k,i}{\prime}={X}_{j,k,i}+{Difference\_Mean}_{j,k,i}$$

where, ${X}_{j,kbest,i}$ is the result of the best student in subject *j*. The teaching factor ${T}_{F}$ specifies how the average should be changed, and ${r}_{i}$ contains a random number ranging from 0 to 1. Also, *X'j,k,i* is the modified value of ${X}_{j,k,i}$. When ${X}_{j,k,i}{\prime}$ provides a superior function value, it is acceptable.

The student phase follows, where each student learns from peers, further refining solutions through the mutual interaction. This interaction encourages diversity in the solution pool, preventing premature convergence to local optima. Students randomly exchange information, adjusting their solutions based on those who have performed better:

6 $${X}_{j,P,i}^{{\prime}{\prime}}={X}_{j,P,i}{\prime}+{r}_{i}\left({X}_{j,P,i}{\prime}-{X}_{j,Q,i}{\prime}\right), if {X}_{total,P,i}{\prime}<{X}_{total,Q,i}{\prime}$$

7 $${X}_{j,P,i}^{{\prime}{\prime}}={X}_{j,P,i}{\prime}+{r}_{i}\left({X}_{j,Q,i}{\prime}-{X}_{j,P,i}{\prime}\right), if {X}_{total,Q,i}{\prime}<{X}_{total,P,i}{\prime}$$

**Figure 2:**
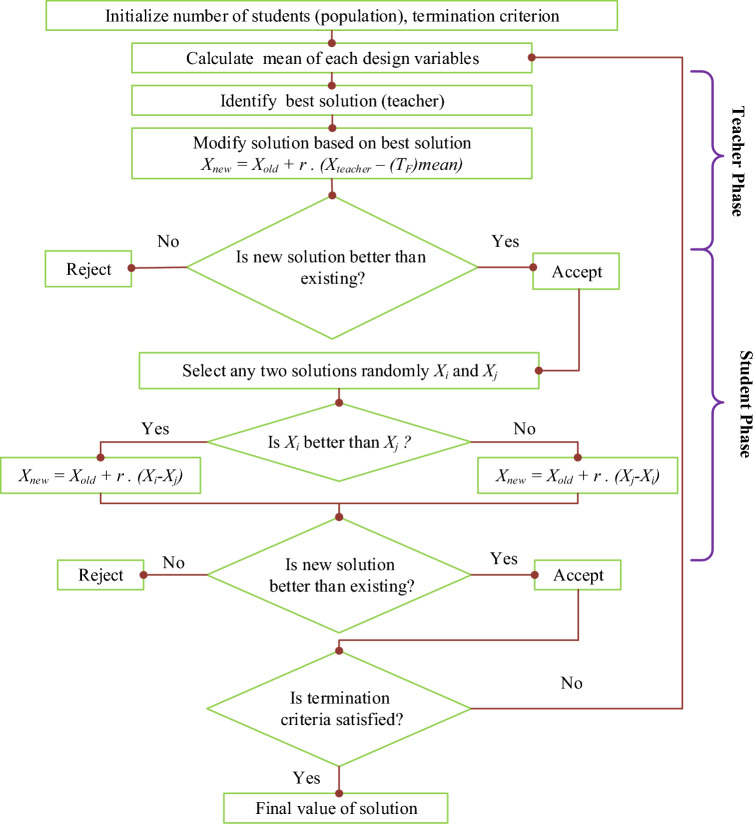
Flowchart for TLBO.

In which ${X}_{j,P,i}^{{\prime}{\prime}}$ is valid as long as resulting in a superior function value^66^. TLBO is noted for not requiring specific algorithmic parameters like crossover rates or mutation probabilities, typically necessary in other evolutionary algorithms. This simplification, along with its dual-phase learning approach, makes TLBO a unique and effective tool for solving optimization problems across various engineering disciplines^66^. The algorithm’s effectiveness lies in its ability to simulate the dynamic and interactive learning environment of a classroom, continuously enhancing the solution quality through collaborative and competitive processes. The TLBO algorithm is illustrated in Fig. 2.

### MGGP

**Figure 3:**
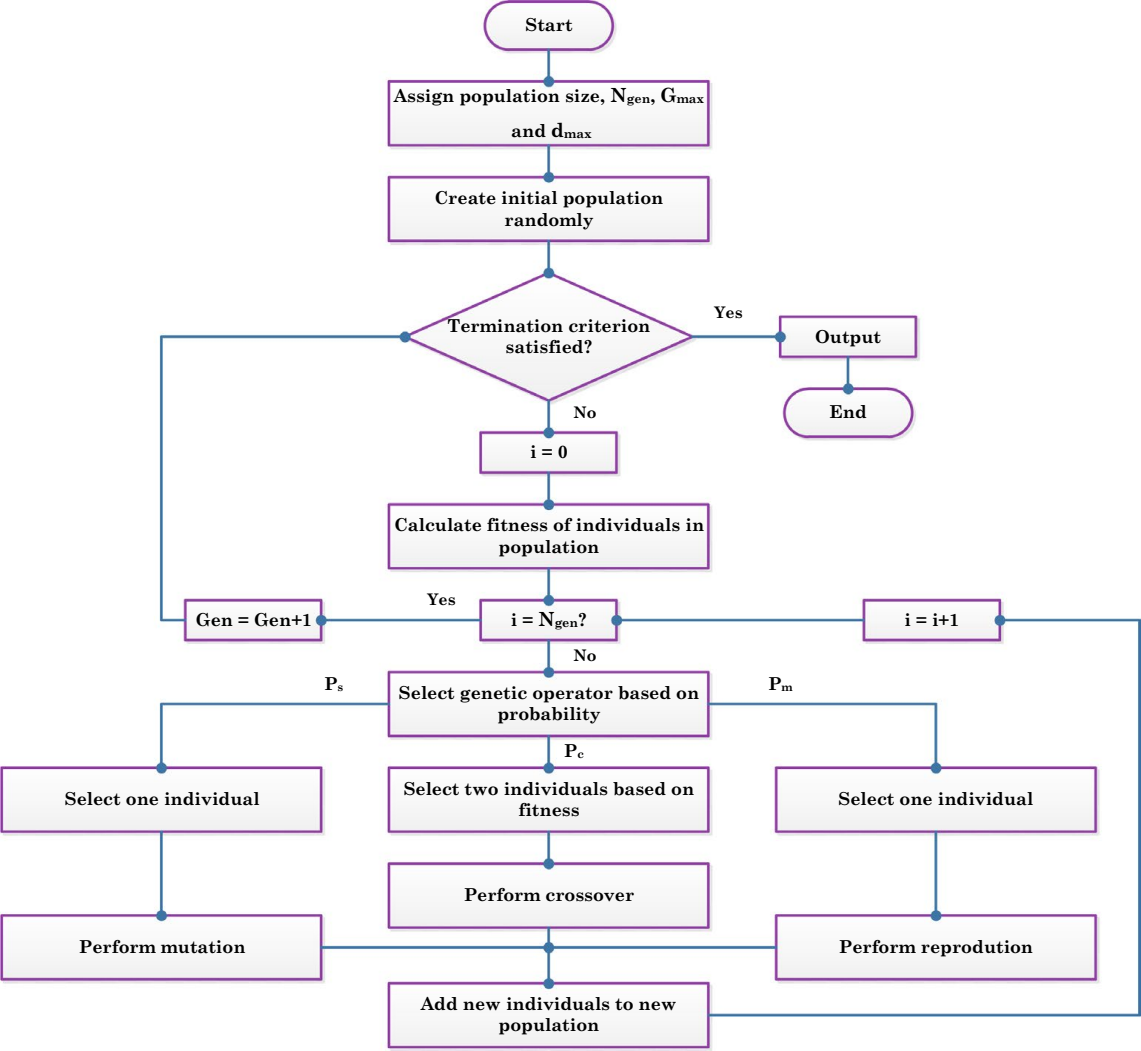
A typical flowchart for an MGGP procedure.

GP, devised by Koza^45^, employs the principles of genetics and natural selection to effectively solve problems across various domains^46,47^. This method uses parse trees, flexible structures that replace fixed-length binary strings, allowing it to adapt to different problem types without being restricted by domain-specific patterns^45,48^. GP operates through a series of evolutionary processes including selection, crossover, and mutation, to optimize solutions based on fitness, which is measured against a predefined error metric^48,67,68^. The flexibility of GP is enhanced by its ability to handle a variety of input variables and functions, termed terminals and primitive functions, respectively, which guide the development of solutions^53^. Moreover, the GP’s effectiveness in producing reliable and diverse solutions is achieved by managing its evolutionary operators, with a particular emphasis on maintaining genetic diversity through mutation to prevent premature convergence^48^. MGGP extends GP by integrating multiple parse trees into a cohesive model, where each tree represents a distinct "gene" contributing to the model’s output^69^. This approach not only allows for the modeling of complex, non-linear relationships but also ensures that the model remains interpretable by maintaining a balance between complexity and parsimony. The MGGP’s ability to combine linear regression with non-linear transformations helps capture intricate interactions within data, providing a robust framework for predictive modeling that is both accurate and efficient^70,71^. This blend of GP and advanced regression techniques makes GP and MGGP powerful tools in the field of computational intelligence, capable of tackling a wide range of engineering and data science problems. Figure 3 shows a typical flowchart for an MGGP procedure.

## Implementation process

### Data collection

**Figure 4:**
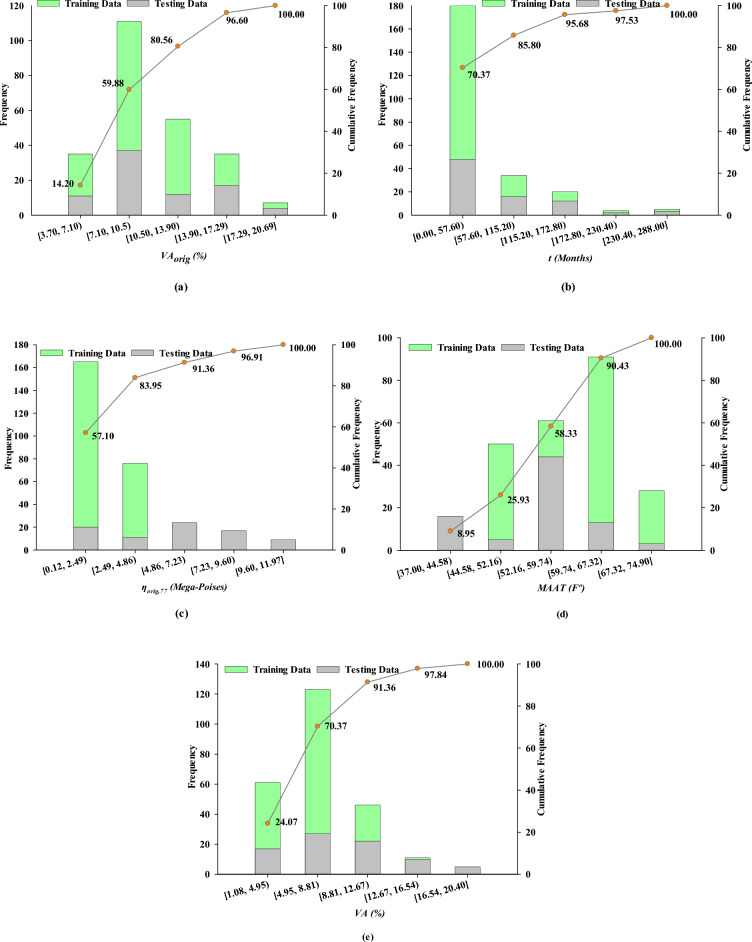
Frequency histogram and cumulative frequency.

The database utilized in this research is from a report by the Transportation Research Board (TRB) of the National Research Council^26^. Among the three parts of the data used in this study, the first comes from GPS-LTPP database, which includes 72 data, the second, contained 177 data collected by Mirza^27^, and the third, derived from NCAT database^26^, which includes 75 data. Further information regarding each of the data sets can be found in the mentioned literature, including project location details, binder grades, or number of sections on each test road. Additionally, in this study, the aged $VA$ (%) was selected as the output of the model, and original as-constructed VA, ${VA}_{orig}$ (%); mean annual air temperature, $MAAT$ (°F); original viscosity at 77 °F, ${\eta }_{orig,77}$ (Mega-Poises); and $time$ (months) were considered as inputs. ${VA}_{orig}$ provides a crucial baseline, allowing for the quantification of changes in VA structure due to traffic and environmental conditions. $MAAT$ is vital for predicting thermal effects on asphalt properties, as temperature significantly impacts the viscoelastic behavior of asphalt. ${\eta }_{orig,77}$ allows for the assessment of the asphalt’s flow characteristics at standard temperatures, influencing how it responds to stress over time. Tracking $time$ enables modeling the evolution of these properties, leading to precise predictions of the pavement performance and maintenance needs. Modeling began with randomly dividing the data into training and testing categories. To accomplish this, 75% of the data were randomly chosen as training data and 25% as testing data. It is worth noting that in Mirza, NCAT, and NCHRP models all data points were used for developing or training of models and no set was employed for validation. Table 1 presents the statistical characteristics of input and output parameters for training and testing data. Figure 4 also depicts the histogram and cumulative frequency for input and output parameters. It can be seen that the amount of ${VA}_{orig}$ is between 3.70 and 20.69%, $time$ varies between 0 and 288 months, ${\eta }_{orig,77}$ falls between 0.12 and 11.97 Mega-Poises, and $MAAT$ ranges from 37.00 to 74.90 °F. Also, the measured VA during the service life changes in the range of 1.14 and 20.40%. A variety of changes in each of the inputs and outputs indicates that the database is diverse, and the models developed over it are generalizable^72^. It should be noted that the original VA depend on the mix design and compaction during construction. Higher initial VA leads to a more noticeable fluctuation in VA compared to lower initial VA. Over time, under various conditions like mix type, traffic, and environment, a final VA value is reached^27^. This final VA is often referred to as "voids at refusal". Heitzman et al.^73^ demonstrated that the as-constructed or original VA in selected LTPP sections alters between 1% to more than 14%.

**Table 1:** Descriptive statistics of training and testing data.

Statistic	${VA}_{orig}$ (%)	$time$ *(months)*	${\eta }_{orig,77}$ *(Mega-Poises)*	$MAAT$ *(ºF)*	$VA$ *(%)*
Training data: 243					
Minimum	3.70	0.00	0.12	37.00	1.14
Maximum	20.69	288.00	11.97	69.50	20.40
Mean	10.15	40.18	1.84	55.33	6.99
Median	9.20	17.00	1.96	56.20	6.60
Standard deviation	3.52	56.27	1.41	6.80	3.11
Skewenss	0.55	1.35	− 0.03	− 0.1	0.66
Kurtosis	− 0.32	1.72	− 1.07	− 0.22	0.22
Testing data: 81					
Minimum	3.80	0.00	0.12	41.80	1.08
Maximum	20.69	240.00	4.13	74.90	15.10
Mean	10.52	49.39	1.91	56.71	6.77
Median	9.80	24.00	1.95	56.90	6.48
Standard deviation	3.65	54.67	1.15	7.16	3.15
Skewenss	0.63	2.03	1.92	− 0.33	0.86
Kurtosis	− 0.26	4.32	11.01	− 0.56	1.42

### Model performance evaluation criteria

Selecting appropriate error measures like R^2^, RMSE, MAE, MAPE, OBJ, and a20-index for evaluating the accuracy and reliability of models in this study was driven by their specific relevance to the objectives and data characteristics^74,75^. R^2^ is used to determine the proportion of variance in the dependent variable that is predictable from the independent variables, making it essential for assessing the model fit. RMSE and MAE provide insights into the average model prediction errors, with RMSE being more sensitive to outliers, thus offering a robust measure of error magnitude^76^. MAPE aids in understanding error in terms of percentage, which is particularly useful for comparisons across different scales or units. The OBJ function combines several statistical measures to provide a comprehensive error summary^77^, while the a20-index offers a unique perspective by focusing on the accuracy within the 20th percentile^78^, crucial for scenarios where smaller prediction errors are critical. Each of these metrics was chosen to ensure a holistic evaluation of the model’s performance, directly supporting the study’s aims to develop reliable predictive models for real-world applications. These statistical indicators are summarized below:

8 $$R^{2} = \left[ {\frac{{\sum\limits_{i = 1}^{N} {(Y_{obs} - \overline{Y}_{obs} )(Y_{pre} - \overline{Y}_{pre} )} }}{{\sqrt {\sum\limits_{i = 1}^{N} {(Y_{obs} - \overline{Y}_{obs} )^{2} } \sum\limits_{i = 1}^{N} {(Y_{pre} - \overline{Y}_{pre} )^{2} } } }}} \right]^{2}$$

9 $$RMSE = \sqrt {\frac{1}{N}\sum\limits_{i = 1}^{N} {(Y_{pre} - Y_{obs} } )^{2} }$$

10 $$MAE = \frac{{\sum\nolimits_{i = 1}^{N} {\left| {Y_{pre} - Y_{obs} } \right|} }}{N}$$

11 $$MAPE = \frac{{\sum\nolimits_{i = 1}^{N} {\left| {Y_{pre} - Y_{obs} } \right|} }}{{\sum\nolimits_{i = 1}^{N} {Y_{obs} } }} \times 100$$

12 $$OBJ = \left( {\frac{{N_{tr} }}{{N_{all} }}.\frac{{RMSE_{tr} + MAE_{tr} }}{{R_{tr}^{2} + 1}}} \right) + \left( {\frac{{N_{tst} }}{{N_{all} }}.\frac{{RMSE_{tst} + MAE_{tst} }}{{R_{tst}^{2} + 1}}} \right)$$

13 $$a20 - index = \frac{m20}{N}$$

in which, ${Y}_{obs}$ and ${Y}_{pre}$ indicate the observed and predicted values, respectively, where the bar signs over the above-mentioned variables represent the average value; $N$ is the number of records; $m20$ is the number of records in which their ${Y}_{obs }/{Y}_{pre}$ ratio falls between 0.80 and 1.20; and $tr$ and $tst$ refer to the training and testing datasets, respectively.

### EPR modeling

The EPR method was used to model the data after it was split into training (75%) and testing (25%) data. This process involved internal functions, including logarithmic, exponential, hyperbolic tangent, and hyperbolic secant. Based on the minimum modeling error, the best model was selected for each function and structure. Also, the presentation type of EPR models was considered in hyperbolic secant, hyperbolic tangent, and logarithmic models as Eq. (14), and in exponential models as Eq. (15). Table 2 outlines other details set for the development of each model.

**Table 2:** Details of tuning parameters for models.

Parameters’ description	Parameters’ tuning			
	Hyperbolic tangent	Hyperbolic secant	Exponential	Logarithmic
Exponent	[− 1,− 0.5,0,0.5,1]	[− 1,− 0.5,0,0.5,1]	[− 1,− 0.5,0,0.5,1]	[− 1,− 0.5,0,0.5,1]
Number of terms	5	5	5	5
Bias	0	0	0	0
Input and output scales	[0 , 1]	[0 , 1]	[0 , 1]	[0, 1]

14 $$Y=\mathit{sum}\left({a}_{i}. {\text{X}}1. {\text{X}}2.f\left(X1.X2\right)\right)+{a}_{0}$$

15 $$Y=sum\left({a}_{i}.f\left(X1.X2\right)\right)+{a}_{0}$$

The representative range mentioned in Table 2 determines the linearity or non-linearity of the model as well as the direct or inverse dependence between input parameters and output parameters.

According to Eqs. (16–19), the optimal models for different functions can be found as follows:

Logarithmic:

16 $$\begin{aligned}VA& = 0.026844\times \frac{MAAT}{{VA}_{orig}\times {\eta }_{orig, 77}}\times Ln\left(\frac{{{VA}_{orig}}^{0.5}}{{\eta }_{orig, 77} }\right)+0.00095733\times \frac{MAAT}{{{VA}_{orig}}^{0.5}}\\ & \quad\times Ln\left(\frac{{VA}_{orig}\times {\eta }_{orig, 77}\times MAAT}{({time+0.0001)}^{0.5}}\right)+0.015524\times MAAT \times Ln\left(\frac{{{\eta }_{orig, 77}}^{0.5}}{{MAAT}^{0.5}}\right) +1.0913\times {VA}_{orig}\\ & \quad+0.14156\times \frac{{VA}_{orig}\times {(time+0.0001)}^{0.5}}{{MAAT}^{0.5}}\times Ln\left(\frac{{(time+0.0001)}^{0.5}\times {{\eta }_{orig, 77}}^{0.5}}{{{VA}_{orig}}^{0.5}\times MAAT}\right)\end{aligned}$$

Exponential:

17 $$\begin{aligned}VA & = 0.00022375\times \frac{(time+0.0001)\times MAAT}{{VA}_{orig}} \times exp\left(0.5\times {VA}_{orig}-0.5(time+0.0001)\right) \\ & \quad + 0.011232\times \frac{{{VA}_{orig}}^{0.5}\times MAAT}{{{\eta }_{orig, 77}}^{0.5}}+3.1933\times \frac{{VA}_{orig}}{{MAAT}^{0.5}}\times exp\left(-0.5\times (time+0.0001)\right)\\ &\quad+0.28873\times {VA}_{orig}\times {{\eta }_{orig, 77}}^{0.5}+1.5509\times \frac{{VA}_{orig}\times (time+0.0001)}{{{\eta }_{orig, 77}}^{0.5}\times {MAAT}^{0.5}}\\ &\quad\times exp\left(-0.5\times (time+0.0001)\right)\end{aligned}$$

Hyperbolic secant:

18 $$\begin{aligned}VA & = \frac{13.9844}{{{VA}_{orig}}^{0.5}\times {(time+0.0001)}^{0.5}{\times {\eta }_{orig, 77}}^{0.5}}\times sech\left(\frac{{{\eta }_{orig, 77}}^{0.5}\times MAAT}{{(time+0.0001)}^{0.5}\times {VA}_{orig}}\right)\\ &\quad+0.008283\times \frac{{VA}_{orig}\times {\eta }_{orig, 77}\times Maat}{{(time+0.0001)}^{0.5}}\times sech\left(\frac{{{\eta }_{orig, 77}}^{0.5}\times {VA}_{orig}}{(time+0.0001)}\right)+2.7264\times \frac{{VA}_{orig}}{{MAAT}^{0.5}}\\ &\quad\times sech\left(\frac{(time+0.0001)}{{{\eta }_{orig, 77}}^{0.5}}\right)+0.58699\times {VA}_{orig}\times sech\left(\frac{{(time+0.0001)}^{0.5}}{{MAAT}^{0.5}}\right)+0.17228{\times {\eta }_{orig, 77}}^{0.5}\\ &\quad\times {VA}_{orig}\times sech\left(\frac{{MAAT}^{0.5}}{{{VA}_{orig}}^{0.5}}\right)\end{aligned}$$

Hyperbolic tangent:

19 $$\begin{aligned}VA & = 0.016025\times \frac{{(time+0.0001)}^{0.5}\times MAAT}{{VA}_{orig}\times {\eta }_{orig, 77}}\times tanh\left(\frac{MAAT}{{VA}_{orig}\times (time+0.0001)\times {{\eta }_{orig, 77}}^{0.5}}\right)\\ &\quad+0.10862\times {{VA}_{orig}}^{0.5}\times {(time+0.0001)}^{0.5}\times {{\eta }_{orig, 77}}^{0.5}\times tanh\left(\frac{{VA}_{orig}\times {\eta }_{orig, 77}}{(time+0.0001)}\right)+0.99502 \\ &\quad \times \frac{{VA}_{orig}}{{MAAT}^{0.5}}\times tanh\left(\frac{1}{{{VA}_{orig}}^{0.5}\times (time+0.0001)}\right)+0.53896\times {VA}_{orig}\times tanh\left(\frac{{MAAT}^{0.5}}{{(time+0.0001)}^{0.5}}\right)\\ &\quad + 0.32525\times {VA}_{orig} \times tanh\left(\frac{{VA}_{orig}}{(time+0.0001)\times {{\eta }_{orig, 77}}^{0.5}\times {MAAT}^{0.5}}\right)\end{aligned}$$

**Figure 5:**
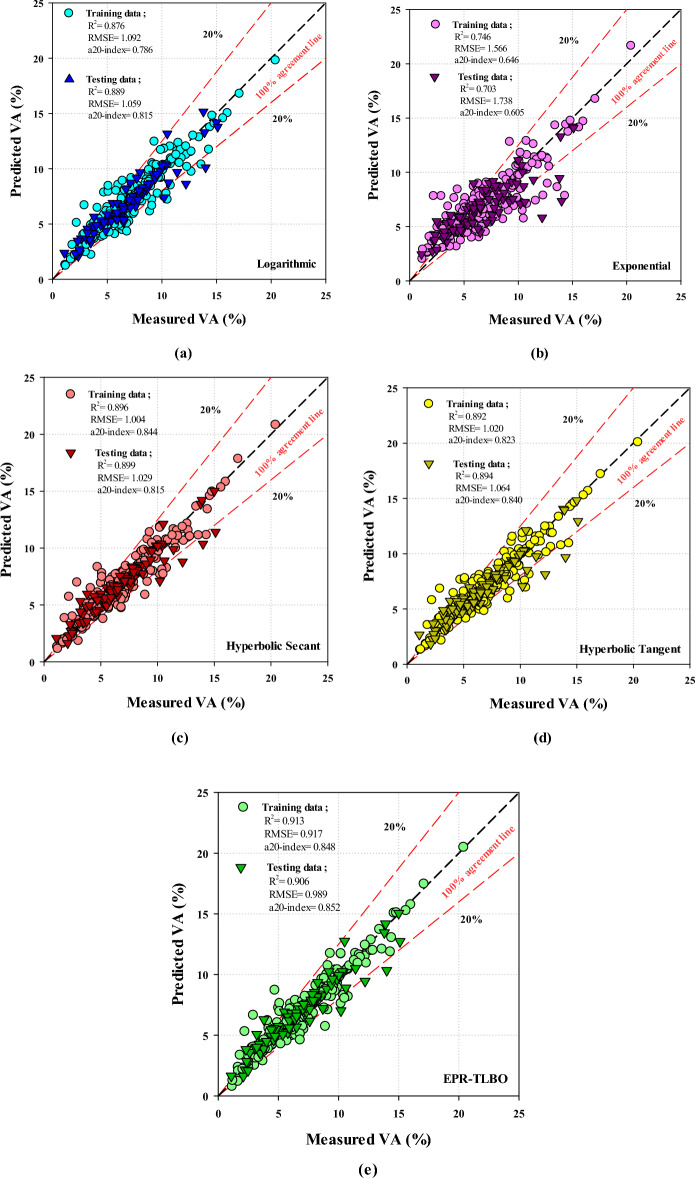
Performance of EPR methods.

In accordance with the training and testing data, Fig. 5a–d demonstrate each EPR model’s ability to predict VA based on measured and predicted values. In a chart, a higher R^2^ value reveals a more efficient model and fewer scattering points than a line of 100% agreement. In comparison with other models, the hyperbolic secant model is selected as the best model based on its highest R^2^ and lowest RMSE values. In the case of the hyperbolic secant model, which was chosen as the best model, the R^2^ values for the training and testing data are 0.8956 and 0.8995, respectively. Also, the RMSE values of 1.0042 and 1.0288, respectively, for the training and testing data, point out an acceptable level of accuracy for the model. An a20-index has been introduced as a new parameter of physical engineering that refers to the number of samples where the difference between the observed and predicted values is less than 20%^79^. Based on Fig. 5, the a20-index equals 0.844 and 0.815 for the hyperbolic secant model in the training and testing phases, respectively. Moreover, in both the training and testing phases, the exponential model gave the worst performance based on a20-index values of 0.65 and 0.61, respectively.

#### Optimizing EPR model with TLBO algorithm

In this study, the TLBO algorithm was employed to improve the validity of the hyperbolic-secant-derived EPR model by fine-tuning constant coefficients, assuming the form of the model is fixed. Accordingly, the objective function was RMSE of the VA values predicted by the TLBO algorithm and the measured values. The stopping condition was applied by reaching the maximum iteration of 1000 and the number of population in each iteration was 50. The equation optimized by the TLBO algorithm is given in Eq. (20):

20 $$\begin{aligned}VA & = \frac{4.0000}{{(time+0.0001)}^{0.195767}{\times {\eta }_{orig, 77}}^{0.120406}}\times sech\left(\frac{{{\eta }_{orig, 77}}^{0.993062}\times {MAAT}^{2.634065}}{{(time+0.0001)}^{3.603024}\times {{VA}_{orig}}^{0790669}}\right)\\ &\quad+0.000779\times \frac{{{VA}_{orig}}^{0.835998}\times {{\eta }_{orig, 77}}^{1.677425}\times {MAAT}^{1.076083}}{{(time+0.0001)}^{0.034285}}\times sech\left(\frac{{{\eta }_{orig, 77}}^{3.343413}\times {{VA}_{orig}}^{1.170881}}{{(time+0.0001)}^{4}}\right)\\ &\quad+0.334897\times \frac{{{VA}_{orig}}^{1.24765}}{{MAAT}^{0.120087}}\times sech\left(\frac{{(time+0.0001)}^{0.554334}}{{{\eta }_{orig, 77}}^{0.076506}}\right)+1.095481\times {{VA}_{orig}}^{0.747923}\\ &\quad \times sech\left(\frac{{(time+0.0001)}^{0.557595}}{{MAAT}^{0.495603}}\right)+1.197353\\ &\quad\times {{VA}_{orig}}^{0.574311}\times sech\left(\frac{{MAAT}^{2.50852}}{{{VA}_{orig}}^{3.837948}}\right)\end{aligned}$$

A number of statistical parameters were evaluated for the overall dataset to assess the precision of the EPR-TLBO model in making forecasts. The results for different statistical parameters are listed in Table 3. Comparing the R^2^ values of EPR-TLBO and hyperbolic secant models reveals that the first model is 1.9% more superior than the second in the training phase, but this superiority drops to 0.78% in the testing phase. Furthermore, RMSEs for the EPR-TLBO and hyperbolic secant models are 0.989 and 1.029%, respectively. MAEs of the EPR-TLBO and hyperbolic secant models are 0.685% and 0.602% in the training phase, respectively, whereas their MAPEs are, respectively, 9.566% and 9.593% in the testing phase.

**Table 3:** Accuracy and performance of each EPR model.

Model	Training				Testing			
	R^2^	RMSE	MAE	MAPE	R^2^	RMSE	MAE	MAPE
Hyperbolic tangent	0.892	1.02	0.663	9.484	0.894	1.064	0.679	10.031
Hyperbolic secant	0.896	1.004	0.685	9.792	0.899	1.029	0.65	9.593
Exponential	0.746	1.566	1.131	16.177	0.703	1.738	1.265	18.677
Logarithmic	0.876	1.092	0.738	10.553	0.889	1.059	0.732	10.806
EPR-TLBO	0.913	0.917	0.602	8.61	0.906	0.989	0.648	9.566

### MGGP modeling

Genetic operators, number of populations, tournament size, number of genes, ERC, crossover, mutation probabilities, and elite fraction are the primary MGGP factors whose rates vary and play an important role in the MGGP algorithm^53,80^. Researchers have proposed^31,81^ that MGGP hyper-parameters must be tuned in a trial-and-error manner to build a VA equation based on ${VA}_{orig}$, $MAAT$, ${\eta }_{orig,77}$*,* and $time$. An optimum setting of MGGP hyper-parameters is obtained through a trial-and-error procedure and by taking into account previous research findings.

As inputs to the model, terminal sets are unrelated variables. As a result, we used trigonometric and arithmetic functions near our problem in the present study. Moreover, the functions of + *, − ,* × *, /, sqrt, exp, Ln, ^2, ^3, 3Rt, sin, cos*, and *tanh* were utilized to design a superior MGGP model. It is necessary to link genes in the multi-genic chromosomes, as well as create large and elaborate gene trees. Here, addition operators (+) are employed to link genes, as these operators give better results than the rest (e.g. − , × , /). Trial-and-error can be used to obtain other MGGP parameters, such as the number of populations, generations, and runs. In the study, 100 populations, 250 generations, and five runs were considered. By changing the number of genes and keeping the other settings parameters fixed, the trial-and-error procedure was followed to select the optimal architecture for the MGGP model. Additionally, the number of nodes was fixed at "*Inf*" so that the MGGP algorithm had more freedom to build models.

**Figure 6:**
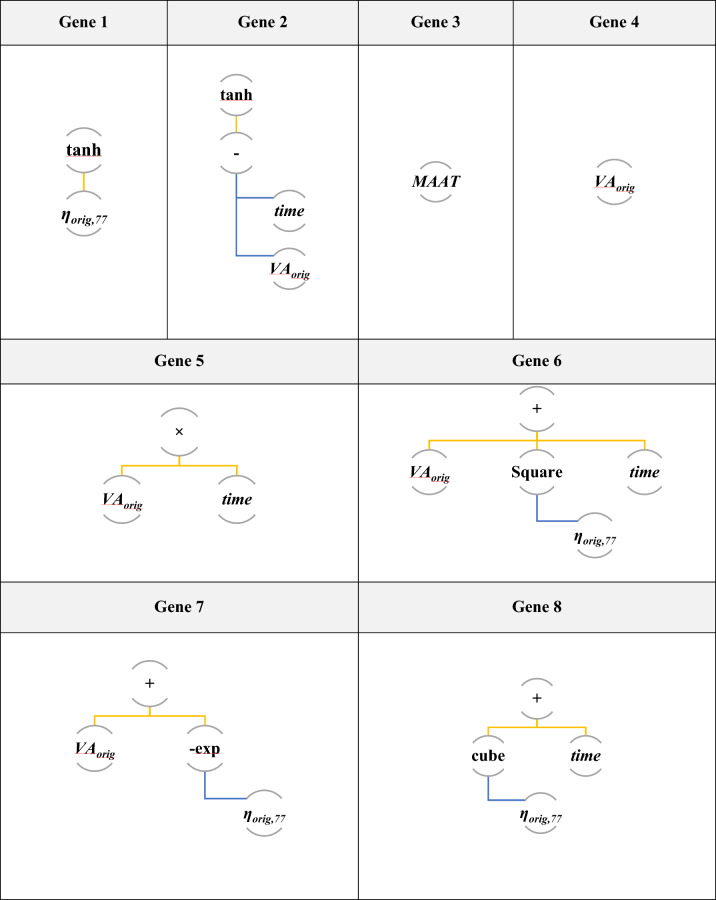

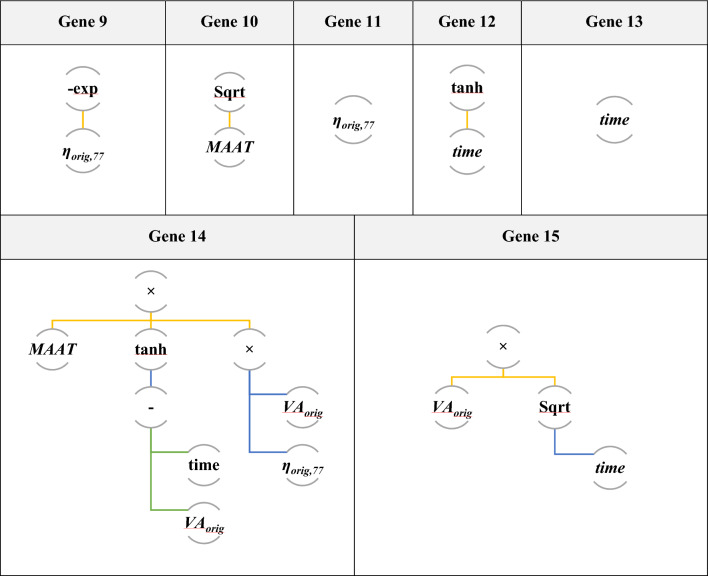
Tree representation of selected fifteenth-genetic chromosome.

Table 4 outlines the performance of various MGGP models with different numbers of genes from 1 to 19. As can be seen, utilizing 15 genes yields the highest testing R^2^ value (0.897). Additionally, the 15-gene model exhibited lower errors in terms of MAE, RMSE, and MAPE values compared to the other models. As shown in Fig. 6, the best chromosome is a tree structure made up of 15 genes connected by addition functions (+). Finally, VA can be predicted with Eq. (21).

**Table 4:** A comparison of various MGGP models for training and testing records.

Number of genes	Training				Testing			
	R^2^	RMSE	MAE	MAPE	R^2^	RMSE	MAE	MAPE
**1**	0.761	1.52	1.183	16.921	0.768	1.527	1.248	18.432
**3**	0.871	1.115	0.799	11.419	0.850	1.220	0.884	13.051
**5**	0.878	1.083	0.731	10.456	0.876	1.117	0.790	11.666
**7**	0.887	1.079	0.725	10.704	0.883	1.061	0.744	10.641
**11**	0.889	1.037	0.720	10.292	0.889	1.048	0.731	10.788
**13**	0.894	1.011	0.698	9.980	0.891	1.024	0.730	10.444
*15	**0.898**	**0.994**	**0.677**	**9.686**	**0.897**	**1.018**	**0.732**	**10.806**
**17**	0.901	0.978	0.680	9.721	0.867	1.151	0.779	11.505
**19**	0.905	0.960	0.652	9.327	0.857	1.188	0.798	11.783

Significant values are in bold.

21 $$\begin{aligned}VA& = 0.00733\times t-0.897\times Maat-0.132\times {VA}_{orig}+2.27\times {\eta }_{orig, 77}+1.29\times ({VA}_{orig})\\ &\quad+0.0439\times {\text{tanh}}\left({VA}_{orig}-t\right)-0.715\times {\text{tanh}}\left(t\right)+8.85\times {\text{tanh}}\left({\eta }_{orig, 77}\right)+19.4\times {\text{exp}}\left(-{\eta }_{orig, 77}\right)\\ &\quad+0.00196\times {VA}_{orig}\times t-0.000818\times ({{\eta }_{orig, 77})}^{3}-0.0887\times {VA}_{orig}\times {t}^{0.5}-0.336\times {VA}_{orig}\\ &\quad\times {\text{exp}}\left(-{\eta }_{orig, 77}\right)+13.4\times {Maat}^{0.5}-0.132\times {{\eta }_{orig, 77}}^{2}-0.000587\times {VA}_{orig}\times Maat\times {\eta }_{orig, 77}\\ &\quad\times {\text{tanh}}\left({VA}_{orig}-t\right)-65.4\end{aligned}$$

**Figure 7:**
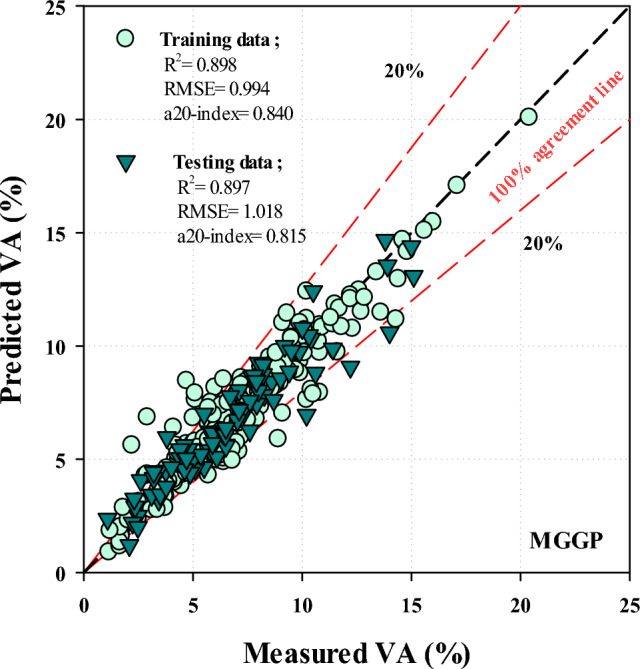
Performance of MGGP method.

Also, the predicted VA values of the MGGP model and measured VA of the training and testing datasets can be seen in Fig. 7. Hence, MGGP is fairly accurate with a20-index values of 0.84 and 0.815 for the training and testing phases, respectively.

**Figure 8:**
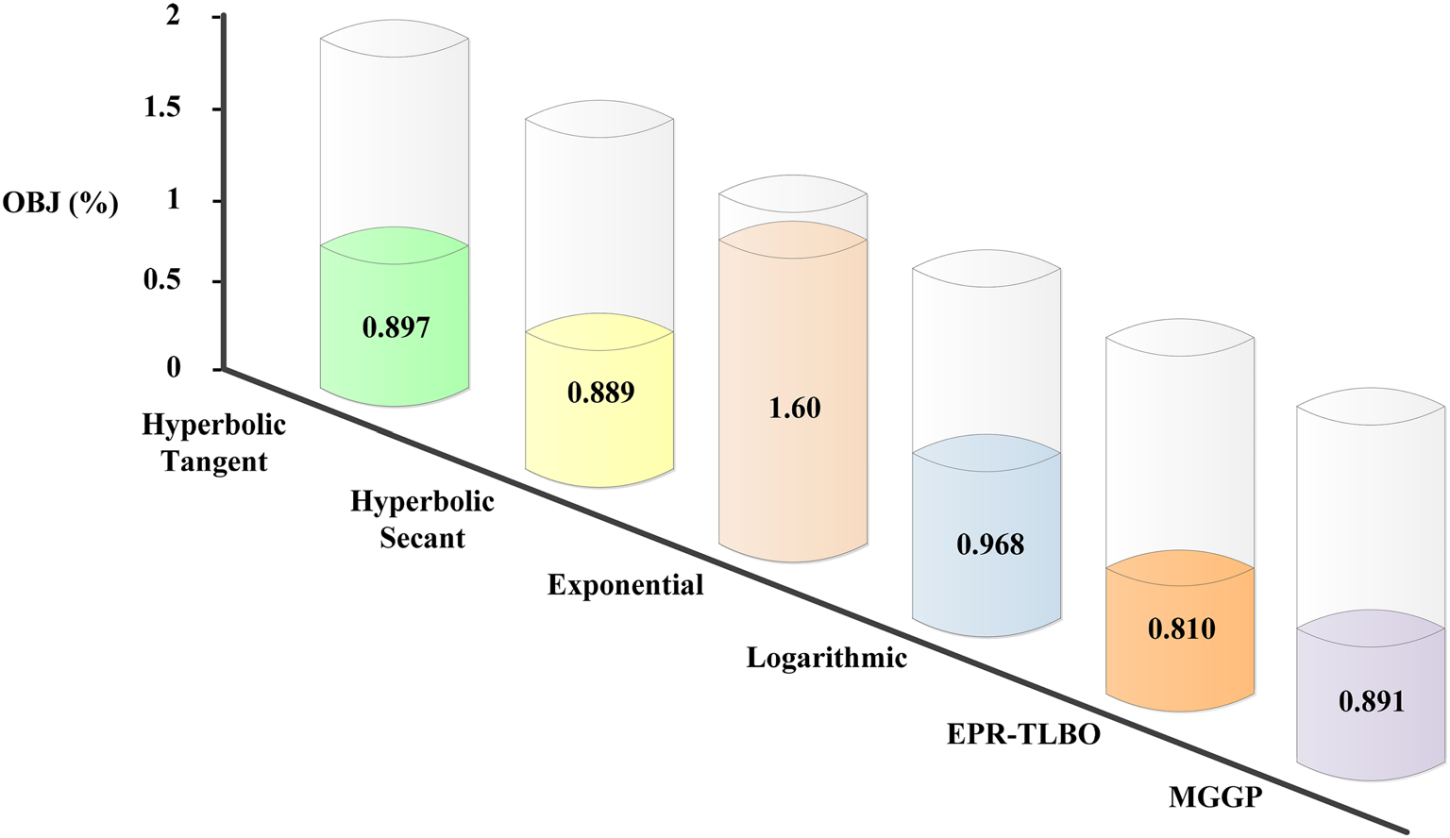
OBJ values of different EPR methods and MGGP.

By combining different statistical indicators for the training and testing datasets, OBJ allows for assessing the performance of a model^82^. When an OBJ high value occurs, it means that a model performs poorly compared to other models^83^. According to Fig. 8, the EPR-TLBO model performs best with an OBJ of 0.81. With an OBJ value of 0.889, the hyperbolic secant model is the next priority, followed by the MGGP model with 0.891.

### Assessment of proposed models compared to existing equations

Table 5 compares the performance of the models proposed in this article with the equations available in the research literature for predicting VA values across all 324 data records. Generally, both developed models in this study were superior to those developed by Mirza, NCAT, and NCHRP. As demonstrated, the best model to predict VA of asphalt concrete is EPR-TLBO model with the highest R^2^ (0.91) and lowest RMSE (0.936%), followed by the MGGP model with an R^2^ value of 0.897 and RMSE value of 1.0%; NCHRP with an R^2^ value of 0.865 and an RMSE value of 1.114%; Mirza with an R^2^ value of 0.8098 and an RMSE value of 1.413%; and NCAT with an R^2^ value of 0.789 and an RMSE value of 1.454%. Calculated MAE and MAPE values signify that the accuracy of the models is prioritized as follows: EPR-TLBO > MGGP > NCHRP > Mirza > NCAT. It should be noted that the performance of the MGGP model in predicting VA values is not far from the EPR-TLBO model.

**Table 5:** Statistical values of developed models.

Model	R^2^	RMSE	MAE	MAPE
Mirza	0.810	1.413	1.023	14.746
NCAT	0.789	1.453	1.070	15.425
NCHRP	0.865	1.144	0.714	10.287
EPR-TLBO	**0.910**	**0.936**	**0.614**	**8.843**
MGGP	0.897	1.00	0.691	9.959

Significant values are in bold.

As a further measure of the validity of the models, the scatter index (SI) as well as the Nash–Sutcliffe efficiency (NSE) coefficient were used:

22 $$SI = \frac{RMSE}{{\overline{Y}_{obs} }}$$

23 $$NSE = 1 - \frac{{\sum\limits_{i = 1}^{N} {(Y_{pre} - Y_{obs} )^{2} } }}{{\sum\limits_{i = 1}^{N} {(Y_{obs} - \overline{Y}_{obs} )^{2} } }}$$

**Figure 9:**
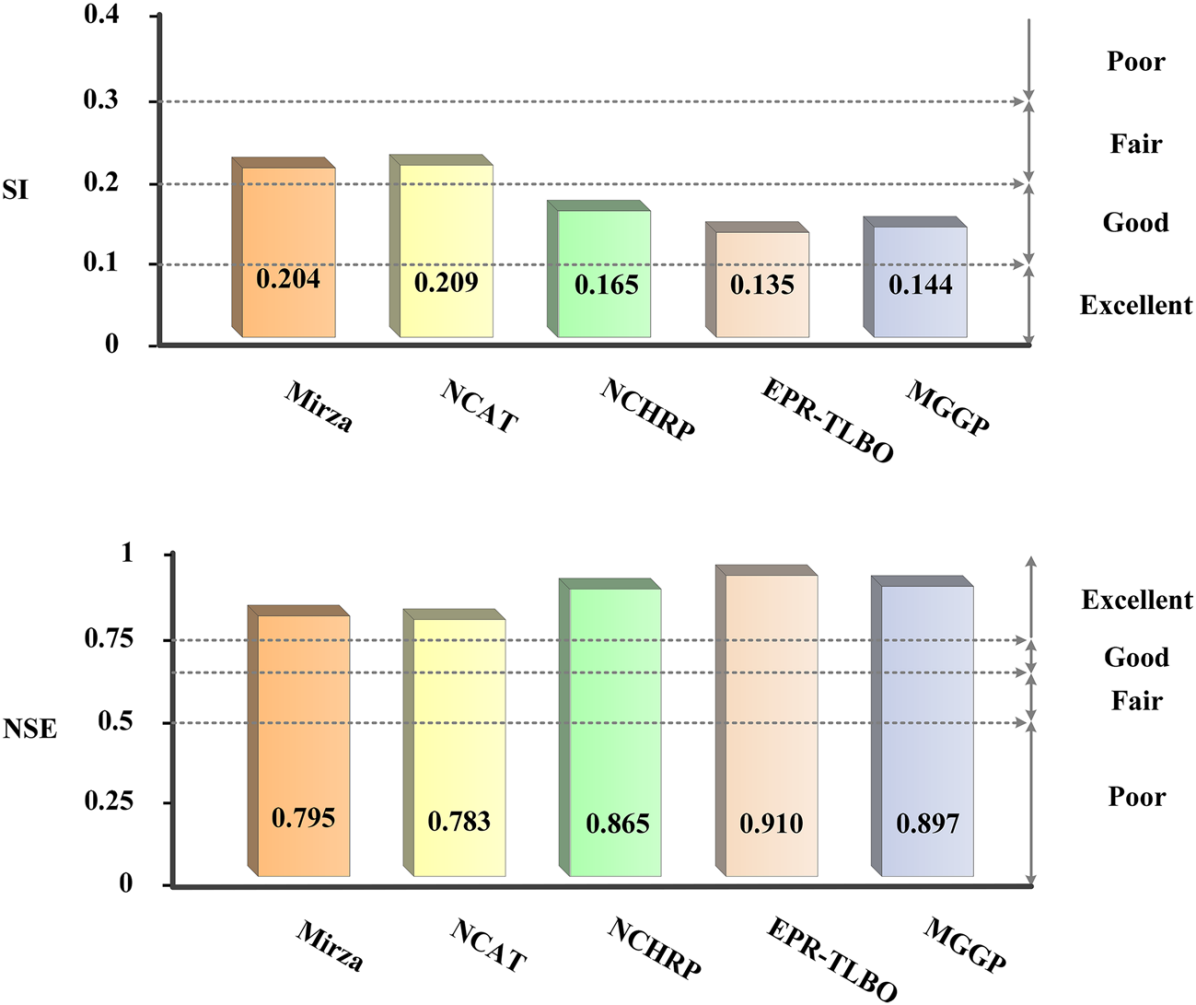
Comparison of SI and NSE calculated values.

Observed values are represented by ${Y}_{obs}$ and predicted values are denoted by ${Y}_{pre}$, while the bar symbols over the respective values illustrate the average value, and N is the total number of records; When the SI value of a model is less than 0.1 or the NSE value is greater than 0.7, the prediction capability of the model is excellent. A model can be considered good if it can achieve SI values between 0.1 and 0.2 or NSE values between 0.65 and 0.75, while if the SI value is between 0.2 and 0.3, or if the NSE value is between 0.5 and 0.65, it can be considered fair, and a model with lower SI and NSE values will perform poorly^72,74^. Based on Fig. 9, the NCHRP, EPR-TLBO, and MGGP models have NSE values above 0.75, indicating excellent learning and generalization accuracy for the NCHRP, EPR-TLBO, and MGGP models. Meanwhile, the NCHRP, EPR-TLBO, and MGGP models have SI values between 0.1 and 0.2, which demonstrates that they are good predictors. The results also reveal that the EPR-TLBO model is more accurate due to having a lower value of SI and a higher value of NSE.

**Figure 10:**
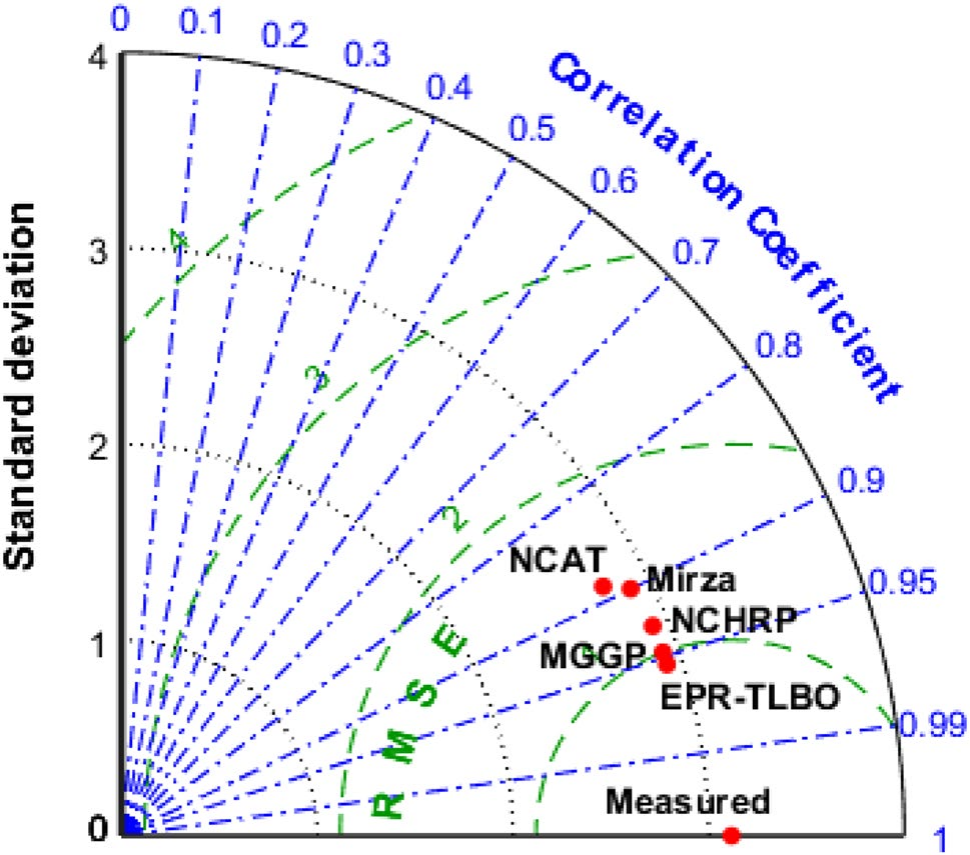
Taylor graph of different model types.

The performance of each model was compared based on the Taylor diagram displayed in Fig. 10. RMSE, R^2^, and STD are statistical parameters used to compare predictions with observations. In the plot, the standard deviation is represented as a circular line connecting the vertical and horizontal axes. Moreover, the horizontal circular green dots demonstrate the RMSE values while the radial blue lines exhibit the R^2^ values. A comparison of the EPR-TLBO model to the observed data reveals that it performs the best. It matches the observed data the closest among all the models.

**Figure 11:**
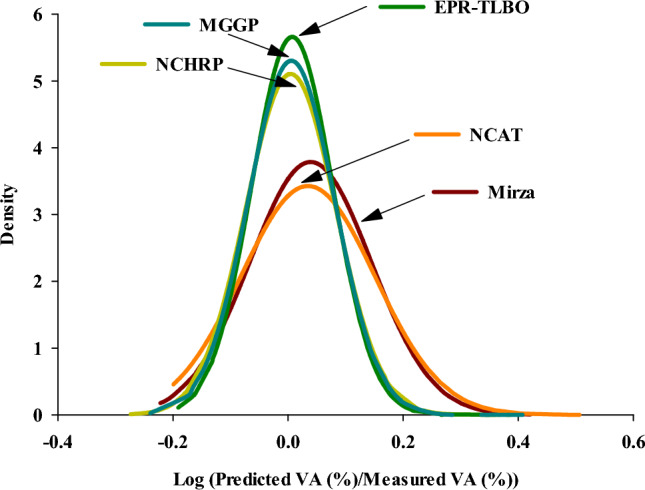
Density functions of different models.

In accordance with Fig. 11, the logarithms of the predicted to measured records ratios for all the VA models for the testing data and their normal density functions were analyzed. Density functions with a higher amplitude show higher accuracy^82^. As can be seen, the maximum density is found close to the zero value of the x-axis for the EPR-TLBO and MGGP models, respectively, highlighting their high testing efficiency.

**Figure 12:**
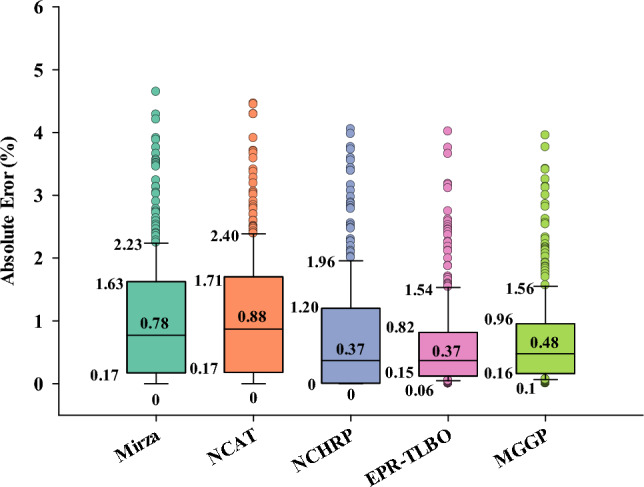
Absolute error boxes of various EL methods.

Figure 12 depicts the absolute error boxes of all the models for the overall datasets. The Mirza, NCAT, NCHR, EPR-TLBO, and MGGP models have interquartile ranges of 1.46, 1.54, 1.20, 0.67, and 0.8%, respectively, underscoring the superior capability of the EPR-TLBO model. Additionally, the median absolute error values of the Mirza, NCAT, NCHRP, EPR-TLBO, and MGGP models are 0.78, 0.88, 0.37, 0.37, and 0.48%, respectively.

## Sensitivity analysis

**Figure 13:**
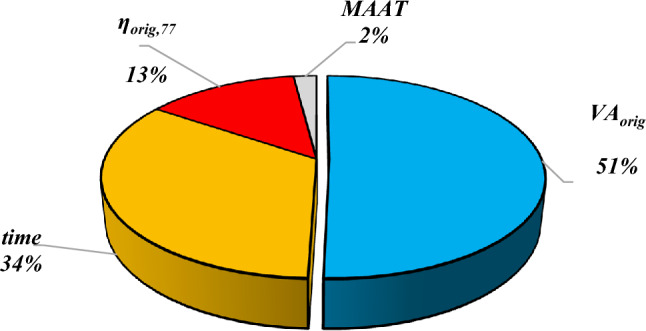
Sensitivity analysis of EPR-TLBO model.

Validating and testing the robustness of the model for unknown data require different analyses of the proposed model. By examining the sensitivity of the input variables, we can determine how the output of a model is affected by the input variables. Here, the sensitivity analysis was conducted according to the method proposed by Gandomi et al.^84^ and Javed et al.^85^. In this method, input variables are calculated as a percentage of the predicted VA through the sensitivity analysis. This calculation is carried out with Eqs. (24 and 25), which are used to determine the sensitivity percentage of VA for any input variable:

24 $${I}_{i}={f}_{max}\left({p}_{i}\right)-{f}_{min}\left({p}_{i}\right)$$

25 $${SP}_{i}=\frac{{I}_{i}}{\sum_{j}^{n}{I}_{j}}\times 100$$

where ${f}_{min}\left({p}_{i}\right)$ and ${f}_{max}\left({p}_{i}\right)$ are the lowest and highest values of the outputs, respectively, calculated from the ${i}^{th}$ input variable, assuming that all other input variables are held constant in their mean values. In SP = 0, the amount of contribution is the lowest, while $SP=100$ represents the highest contribution. Figure 13 features the results of the sensitivity analysis of the input variables for the EPR-TLBO model. As can be seen, ${VA}_{orig}$ (%) and $MAAT$ *(ºF)* provide the largest and smallest contributions to the asphalt concrete mixture VA, respectively. Overall, the importance of features can be prioritized as  ${VA}_{orig}$ (%) > $time$ *(months)* > ${\eta }_{orig,77}$ *(Mega-Poises)*> $MAAT$ *(ºF)*. This finding is consistent with the results of the sensitivity analysis for the NCHRP model^26^. The graphical sensitivity analysis employing the NCHRP model revealed that two parameters ${VA}_{orig}$ and *time* strongly affect the VA value, and the $MAAT$ parameter has a much smaller effect on VA during the service life^26^.

## Parametric study

**Figure 14:**
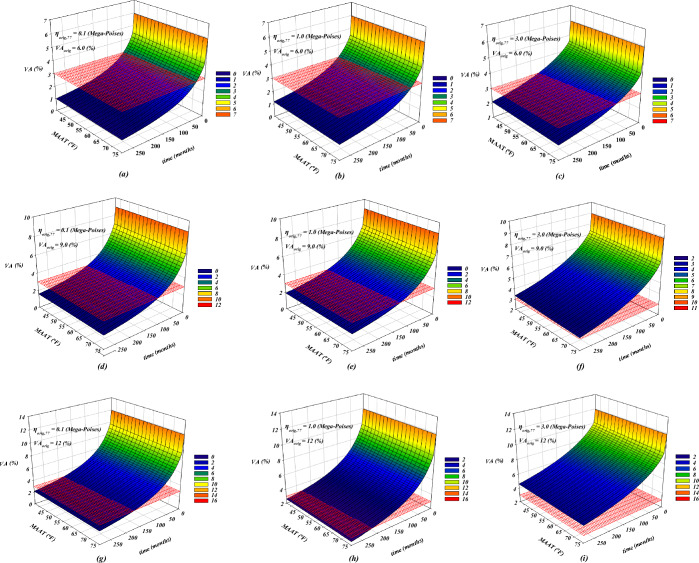
Analysis of impact of input variables on aged VA of asphalt concrete mixtures.

The life expectancy of conventional road pavement is between 20 and 30 years^86^. Asphalt concrete pavements are commonly damaged by fatigue, along with thermal cracking and rutting^87^. The longitudinal depression along the wheel path, called rutting, usually has upheavals on both sides^88^. Due to the high as-constructed VA content of the material, it is expected that VA will reduce over time, causing rutting during initial traffic periods^88^. Eventually, the material will undergo shear flow when it reaches its densest state with optimum aggregate interlock or refusal VA content, typically between 2 and 3%^89^. Moreover, there should be enough flexibility in the asphalt to resist distress. When asphalt-coated aggregate particles are compacted, they become stable and resistant to various types of deformation while improving the mixture’s durability and reducing its permeability^90^. Several researchers concluded that dense-graded asphalt mixes should not contain less than 3.0% VA during the service life due to subsequent pavement distresses^91–94^. Based on the previous studies, adding just 1.0% more VA to the asphalt mix may result in a 35% decreased pavement fatigue life and doubled permeability^95^. Also, an increase of 1.0% in VA over the base level of 7.0% tends to reduce the pavement life by 10%^96^. Thus, engineers and road designers can make accurate and cost-effective decisions by having a fairly accurate estimate of the service life of asphalt pavement in exchange for different influencing variables such as ${VA}_{orig}$ (%), ${\eta }_{orig,77}$ (Mega-Poises), and $MAAT$ (°F). However, researchers are reluctant to conduct experimental studies because of time constraints and the cost of preparing numerous asphalt core samples and doing lab tests. With the development of prediction models, parametric analysis can also be performed along with determining how input variables affect output. To achieve this, two parameters of $time$ and $MAAT$ were considered as being between the minimum and maximum levels in Fig. 14, and other parameters such as ${\eta }_{orig,77}$ of 0.1, 1.0, and 3.0 Mega-Poises, and ${VA}_{orig}$ of 6, 9, and 12% were assumed as constants. After that, VA was calculated according to the desired changes in parameters. In a general view, the VA percentage is negatively correlated to *time* and $MAAT$, whereas ${VA}_{orig}$ and ${\eta }_{orig,77}$ are directly related to the VA amounts. To find an expectation for the service life of asphalt concrete, a plane with a constant VA value of 3.0 cut the main mesh surface. As a result, the intersection of these surfaces determines the approximate service life of asphalt concrete. In Table 6, low and high $MAAT$ *s* of 37 and 75 °F are compared with their respective calculated service life. At ${\eta }_{orig,77}$ of 0.1 Mega-Poises, increasing ${VA}_{orig}$ from 6 to 9 and 12% adds 70 and 140 months to the asphalt concrete service life equally at the minimum and maximum $MAAT$ *s*, respectively. Also, at ${\eta }_{orig,77}$= 1.0 Mega-Poises, a 1.5-fold increase in ${VA}_{orig}$ extends the service life by 2.8 to 3.1 times at $MAAT$ of 37 and 75 °F, respectively. Meanwhile, when ${VA}_{orig}$ doubles, at different $MAAT$ *s*, the pavement serviceability takes 4.6–5.4 times longer. In the case of ${\eta }_{orig,77}$ = 3.0 Mega-Poises, for ${VA}_{orig}$ higher than 6%, the study was unable to provide a pavement life expectancy estimate. Table 6 enables a nuanced analysis, allowing designers to assess how various factors might influence the longevity and durability of asphalt under typical service conditions. By integrating these parameters into their evaluations, designers can make more informed decisions regarding material choices and construction strategies, ultimately enhancing the performance and sustainability of pavement installations. In the analysis of the service life, only the rutting life and bleeding of asphalt mixture have been considered, and other failures such as fatigue damage, moisture sensitivity, and aging due to high VA have not been taken into account.

**Table 6:** An overview of parametric study′s results.

Parameter			Service life (month)
${VA}_{orig}$ *(%)*	${\eta }_{orig,77}$ *(Mega-Poises)*	$MAAT$ *(ºF)*	
6	0.1	37	40
6	0.1	75	30
6	1	37	50
6	1	75	40
6	3	37	120
6	3	75	90
9	0.1	37	110
9	0.1	75	100
9	1	37	140
9	1	75	125
9	3	37	Above 288
9	3	75	Above 288
12	0.1	37	180
12	0.1	75	170
12	1	37	230
12	1	75	215
12	3	37	Above 288
12	3	75	Above 288

## Limitations

The current limitations in the data and methodology for the pavement performance modeling are largely due to insufficient comprehensive datasets, particularly lacking in detailed traffic load and environmental data. To enhance future research, there is a need for more robust data collection systems, incorporating real-time traffic monitoring and environmental conditions, alongside more frequent pavement condition assessments. Additionally, integrating advanced machine learning techniques like deep learning, adding more predictive variables such as material properties and maintenance history, and employing rigorous model validation strategies can remarkably improve the accuracy of these models. By expanding the depth and breadth of data and employing cross-disciplinary approaches, future studies can develop more precise and reliable predictive models, thus enhancing decision-making in pavement management and extending the service life of pavement infrastructures. In order to specify the limitations of the dataset used in this study, the database specifications including the number of data points and the range of independent variables are listed in Table 7.

**Table 7:** Characteristics of dataset for developing models in this study.

Data point	${VA}_{orig}$* (%)*		$time$ *(months)*		${\eta }_{orig,77}$* (Mega-Poises)*		$MAAT$* (*^*o*^*F)*		*VA (%)*	
	Min	Max	Min	Max	Min	Max	Min	Max	Min	Max
324	3.7	20.69	0	228	0.12	11.97	37	74.9	1.08	20.4

## Conclusions

This article aimed to predict VA values of asphalt concrete mixtures throughout their service life by adopting a hybrid EPR approach with the TLBO algorithm and GP. As part of this effort, 324 data from the literature have been collected regarding VA during the service life. As input variables, we selected ${VA}_{orig}$ (%), $time$ (months), ${\eta }_{orig,77}$ (Mega-Poises), and $MAAT$ (°F). Below is a summary of the findings:

- TLBO has shown to be capable of optimizing the EPR coefficients in an efficient manner.
- In the testing and training phases, the EPR-TLBO model is superior to the GP model according to statistical indicators. All of the proposed models in this study were correctly trained and all of their predicted values were highly correlated with low error rates, based on a comparison between the EPR-TLBO and GP models with those from the literature that was conducted in this study.
- The EPR-TLBO and GP models were able to predict over 80% of records with a maximum deviation of 20% from the actual results.
- The newly developed models exhibited excellent performance with SI between 0.1 and 0.2 and NSE greater than 0.75.
- A comparison of the estimated values and their correlation with the observed values suggests that the predictions of the EPR-TLBO model are closer to those of Mirza, NCAT, and NCHRP. A high OBJ value indicates poor existing model’s performance over those proposed in this article.
- As determined by the sensitivity analysis, ${\eta }_{orig,77}$ (Mega-Poises) is the most significant of the four input variables.
- The EPR-TLBO models have the superior capability when compared to other existing models because of the interquartile ranges of absolute error boxes equal to 0.67%.
- The highest normal density function values were found near the zero point of the logarithms of the predicted to measured results ratios for both the EPR-TLBO and GP models, respectively, highlighting their remarkable testing efficiency.
- A parametric study revealed that regardless of $MAAT$, ${\eta }_{orig,77}$ of 0.3 Mega-Poises and ${VA}_{orig}$ above 6.0% can be ideal for improving the pavement service life. It was also observed that with an increase of $MAAT$ from 37 to 75 °F, the serviceability of asphalt concrete takes 15 months less on average.
- EPR-TLBO, due to its robust optimization capabilities, may excel in complex scenarios but can be prone to overfitting if not properly regulated. MGGP, offers a flexible model structure that can adaptively fit diverse data patterns, but it may also become overly complex, making it difficult to interpret. Traditional regression models, while generally easier to understand and implement, might lack the nuanced fitting capabilities of more advanced algorithms, potentially leading to under fitting in complex datasets. Each model’s performance thus reflects a trade-off between accuracy and model interpretability, highlighting the importance of selecting the right model based on specific project requirements and data characteristics.
- The new models proposed in this study allow for more accurate predicting of VA of asphalt concrete during the service life. More accurate predicting of VA during the service life results in more accurate predicting of dynamic modulus change during the service life which is crucial for the pavement analysis and design for both reconstruction and rehabilitation projects. Also, accurate models for predicting VA of asphalt concrete during the service life are beneficial to detect the premature damage of asphalt concrete layers related to low VA such as bleeding and rutting.

## Data Availability

Data associated with the present study will be available on request from the corresponding authors, Ali Reza Ghanizadeh (ghanizadeh@sirjantech.ac.ir) and Alireza Bahrami (alireza.bahrami@hig.se).
